# The impact of digital skills on the mental health of rural residents: from the perspective of happiness

**DOI:** 10.3389/fpsyg.2025.1471488

**Published:** 2025-07-11

**Authors:** Lei Zhou, Yubo Peng, Qing Xia

**Affiliations:** ^1^Zhejiang Shuren University, Hangzhou, China; ^2^Zhejiang Academy of Commerce, Hangzhou, China

**Keywords:** digital skills, rural residents happiness, material compensation, emotional immersion, digital dividend

## Abstract

Happiness is one of the important manifestations of the psychological health of rural residents. Based on the data of the 2020 China Rural Revitalization Comprehensive Survey (CRRS), this paper constructs a theoretical analysis framework of “material compensation-emotional immersion” and adopts OLS model and instrumental variables method to study the influence of digital skills on the happiness of rural residents and the underlying mechanism. The regression results of the OLS model show that there is an “inverted U” curve relationship between digital skills and rural residents' happiness, which verifies the happiness effect of digital skills mastery, and also reveals that as the level of digital skills increases, it is easy to lead to distorted upward comparisons, fall into digital traps and form digital addiction, resulting in lower happiness. The mechanism analysis shows that the “inverted U” effect of digital skills on the happiness of rural residents is mainly transmitted through the material compensation effect, i.e., the objective income and subjective income satisfaction first rise and then fall. Although digital skills gradually build up a positive affective immersion effect, i.e., an increase in trust, it is not sufficient to offset the negative effect of the material compensation effect. Heterogeneity analysis shows that the happiness effect of digital skills is present in both low- and high-income farmer groups, reflecting the universality of the digital dividend. However, farmers at high income levels are more negatively affected, showing an “inverted U” type non-linear relationship, while farmers at low-income levels are only positively affected, showing a positive linear relationship. In addition, digital skills can reduce the welfare gap between rural residents with different levels of education. Accordingly, this paper provides empirical evidence on how to bridge the digital divide in the process of digital village construction in order to better improve the happiness of rural residents.

## 1 Introduction

The familiar traditional idioms of “a little wealth is peace” and “happy farmers and disillusioned achievers” seem to indicate that Chinese farmers seem relatively happy despite their poverty and low socioeconomic status (Liao, 2014). A possible explanation for this is provided by an economic study of subjective wellbeing^1^ in rural China: the basic reason why most rural residents in China are happier is that they have a limited information set and a narrow reference group (Knight et al., 2009). This explanation may no longer be tenable in the digital age, when China's rural society is gradually shifting from an “acquaintance society” to a “semi-acquaintance society” as a result of the impact of digital technology access. Scholars have already conducted studies on related topics, all of which concluded that digital technology has a happiness effect on rural residents (Yin et al., 2021; Luo and Liu, 2022). However, in reality, this is based on digital technology access, and the happiness effect of the digital technology use gap remains to be proved.

The access gap and the usage gap of digital technologies correspond to the “primary digital divide” and the “secondary digital divide”, respectively (Bowen and Morris, 2019). The development of digital technology is often accompanied by the emergence of a “digital divide” between regions and groups, especially in rural areas (Chen and Zhou, 2022). With the implementation of information projects such as the village-to-village connection and broadband to the countryside, the “primary digital divide” has been gradually bridged, however, due to the weak information application and identification ability of rural residents, there is still a large “secondary digital divide”^2^ (Pesci et al., 2023). As a result, digital skills have become an important factor in the wellbeing of rural residents. Digital skills in this paper are understood in a broad sense, referring to the ability to apply digital technologies to engage in behaviors such as transactions, socializing, and entertainment. So do digital skills still have a happiness effect? And how do digital skills affect changes in the wellbeing of rural residents? The above questions are very important but do not seem to have been discussed by scholars. The report of China's 20th Party Congress also emphasized the need to build a livable, workable and beautiful countryside, which, together with the current strategy of building a digital countryside, makes it relevant to study the relationship between digital skills and the wellbeing of rural residents.

In response to the above issues, this paper attempts to make marginal contributions in the following three aspects: Firstly, research perspectives. In view of the gradual transition from the “first-level digital divide” to the “second-level digital divide” within rural society, this paper intends to further explore the issue from the perspective of digital skills. Secondly, indicator measurement. The measurement of happiness indicators has gradually changed from single-variable to multi-dimensional indicators, but it mostly takes into account the subjective self-assessment of the past or current living conditions, and does not take into account the long-term happiness, so this paper intends to add the variable of “future life expectation” to provide a more detailed portrayal of the sense of wellbeing. Thirdly, research data. Relying on data from the China Rural Revitalization Comprehensive Survey (CRRS), which is oriented toward academic and policy research on rural revitalization, it reflects the basic situation of China's rural revitalization in the new era more practically and comprehensively than traditional survey data, and has the advantages of being scientific, timely and comprehensive.

## 2 Changes in wellbeing in social transition: a literature review

The study of happiness has evolved significantly since Easterlin's (1974) seminal work on the wealth-happiness paradox. While early scholarship predominantly focused on economic determinants—including debates around income growth (Jebb et al., 2018), inequality (Ngamaba et al., 2018), and transparency (Perez-Truglia, 2020), subsequent research has progressively revealed the multidimensional nature of wellbeing through three interactive analytical lenses.

At the individual level, demographic factors interact dynamically with behavioral patterns, where gender-specific emotional responses to migration (Jiang et al., 2012) and religion-mediated conduct (Campante and Yanagizawa-Drott, 2015) exemplify how personal characteristics moderate subjective wellbeing. Household dynamics present a paradox of material and relational influences, where economic indicators like housing equity (Napa et al., 2020) and debt (Ram et al., 2020) coexist with non-economic factors such as parental care (Su et al., 2013) and gender-based family composition (Li, 2021), suggesting complex compensation mechanisms between financial security and emotional fulfillment. Societal factors further complicate this matrix through dualistic labor market effects (Aghion et al., 2016) and environmental stressors (Ludwig et al., 2012; Anderson et al., 2016), revealing how macro-level forces simultaneously enable and constrain individual happiness.

The digital revolution introduces new dimensions to these established frameworks, particularly in rural China where internet penetration reconfigures traditional happiness determinants. Current research bifurcates into two conflicting paradigms: The enhancement perspective emphasizes ICT's capacity to strengthen class identity through social mobility (Luo and Liu, 2022), a mechanism supported by socioeconomic status elevation (Zhao, 2012) and enriched social connectivity (Epley et al., 2022). Conversely, the impairment thesis highlights psychological costs from upward social comparisons (Bao et al., 2021; Lohmann, 2015) and digital addiction pathologies (Tso et al., 2022), exposing fundamental tensions between information accessibility and emotional wellbeing.

To summarize, previous literature has been richly researched around the connotation of happiness and the impact of digital technology on happiness, which lays a solid foundation for the study in this paper. However, further research can be done in the following two points: Firstly, Internet access shows both positive and negative effects on wellbeing, and based on this paradoxical point, no literature has yet analyzed it from a more in-depth perspective, i.e., the digital skills perspective. Secondly, for the wellbeing of rural residents, digital technology can affect them both materially and spiritually, but there is little literature that discusses the combination of both material and spiritual impacts. These limitations are areas where this paper can be further expanded.

## 3 Theoretical analysis and research hypotheses

Diener et al. (2018) argue that subjective wellbeing will be realized when physiological and psychological needs are met. Digital technology is able to satisfy the physiological and psychological needs of rural residents to a certain extent, as demonstrated by the positive effects shown in rural residents' income (Leng, 2022), income gap (Zhang et al., 2023) and social identity (Liu et al., 2023). In addition, the direct impact of digital technology in enhancing rural residents' sense of wellbeing has also been demonstrated (Leng and Zhu, 2018; Yin et al., 2021; Luo and Liu, 2022). The existence of the “secondary digital divide” has led to the inability of rural residents to enjoy digital dividends, which has become an important factor limiting the ability of digital technology to enhance happiness. Therefore, the acquisition of digital skills has become one of the most important abilities for rural residents to apply digital technology to increase their wellbeing, satisfy their physical and psychological needs, and enhance their sense of wellbeing (Chen and Zhou, 2022). Notably, the expansion of the breadth of digital skills enhances wellbeing through the cumulative effect of resources and the mechanism of complementary capabilities. According to Sen's Capability Approach, each digital skill represents instrumental freedom to access specific digital resources, and the superposition of skill types can break through the limitations of a single technology application to form a synergistic network of digital resources. Existing research reveals that the welfare effect of digital skills is significantly breadth-dependent, and that the diversity of skill types expands the scope of digital dividend capture through the synergistic effect of resources (Luo and Zhao, 2022), farmers who use both mobile payment and social media have higher incomes than single-skill users (Leng, 2022).

It cannot be avoided that digital skills may have a negative impact on the wellbeing of rural residents. The phenomenon of digital addiction has gradually spread to rural areas, and it is commonly believed that the negative impact of digital technology on wellbeing stems from the crowding out of offline social activities and traditional channels of information access (Luo and Liu, 2022). In fact, new media have not substantially replaced socializing with family and friends and other social activities (Hampton and Shin, 2022). It is all the more important to be wary of the algorithmic capture^3^ of rural left-behind children by online games and short videos, as well as the toxic effects of online lending, betting and scams on rural residents. The addictive economy that comes with digital skill acquisition instead, and the economic loss that comes with online disinformation, both of which greatly reduce the wellbeing of rural residents. This leads to the first research hypothesis.

**Hypothesis 1:** The impact of digital skills on rural residents' happiness is in an “inverted U” shape, that is, the mastery of digital skills can obtain a “digital dividend” and thus increase happiness, but as the level of digital skills increases, it is easy to fall into “digital traps”, which in turn reduces the sense of happiness.

This section further attempts to provide some explanations for the causes of Hypothesis 1, that is, the mechanism that shapes the “inverted U” curve relationship between digital skills and the wellbeing of rural residents. In conjunction with the literature review, research on digital technologies has essentially identified two sets of motivations that can influence the wellbeing of rural residents. The first set of motivations can be summarized as material compensation factors, which emphasize the material benefits of access to information, reduced transaction costs, and entrepreneurial support brought about by digital technologies, and which can essentially be attributed to the impact on income. The second group of motives can be summarized as emotional immersion factors, which emphasize the continuous impact of emotional contagion and digital addiction, among others, on emotions. The two groups of motives reflect individuals' external economic behaviors and internal psychological processes; therefore, this section will theoretically analyze the mechanism of the impact of individual farmers' digital skill mastery level on wellbeing through two aspects, and propose corresponding research hypotheses on this basis.

### 3.1 Material compensation effects

Firstly, the acquisition of digital skills helps farmers to enjoy the “digital dividend” at an early stage and increase their income. According to the theory of decision-making behavior of farmers, farmers are rational economic people who seek to maximize profits under specific resources, information and technology conditions; when the constraints are strict, farmers can only make production or employment decisions under limited conditions; when the constraints are relaxed, farmers are able to make the optimal allocation of the existing factors of production, or make the most satisfactory choices. And an increase in the level of digital skills, which implies an increased ability to access and apply information, relaxes constraints (Tack and Aker, 2014) and is supposed to boost farmers' incomes.

This is manifested in three ways: (1) Increasing operating income. Digital skills help farmers master new agricultural technologies in a timely and effective manner and improve agricultural productivity (Kaila and Tarp, 2019; Giulivi et al., 2022), while timely insights into market and commodity information can reduce risks associated with agricultural production and marketing. As a result, agricultural business income is increased. The acquisition of online trading skills, such as rural e-commerce, can also stimulate farmers' entrepreneurial activities and increase non-farm operating income (Zhou et al., 2020). (2) Increasing wage income. Digital skills can help farmers quickly and accurately access job market information, such as job vacancies and job requirements, and strengthen employment selectivity and matching, which helps rural laborers move to non-farming industries; with the low-cost advantage of online education, human capital can be upgraded in a short period of time, and employment stability can be strengthened, thus increasing wage income. (3) Increased property income. As farmers participate in digital life and production, digital financial platforms and mobile payment methods have greatly lowered the threshold for financial investment, expanding the scale of farmers' financial assets and increasing their property income. In addition, the sweetness of the “digital dividend” can, to a certain extent, enhance farmers' subjective income satisfaction.

However, as the level of digital skills increases, the lack of supervision and guidance within rural areas makes it easier to fall into “digital traps”, leading to a reduction in income. On the one hand, digital addiction can jeopardize social, sleep and physical and mental health, greatly affecting school and work; on the other hand, it is easy to fall into gambling and betting, online game trading and false part-time jobs and other online scams, and suffer huge economic losses. An opinion poll shows that up to 90% of people who are victims of Internet fraud are rural parents living alone.^4^ Among them, “health care video”, “recharge to send cell phones”, and “free to receive red packets” are the most vulnerable scenarios; “online shopping”, “short video live”, and “online KTV” is the most vulnerable platform. Stronger digital skills mean being able to dabble in a wide range of apps using a smartphone, making it easier to fall for online scams. In addition, as the level of digital skills improves, access to information becomes wider, and reference groups are no longer limited to residents of the village or people they know, upward comparisons make traditional concepts of life suffer. In particular, today's social apps and short-video apps are full of false displays of wealth, which can lead to distorted upward comparisons, making people feel that their lives are not as good as they want them to be and leading to a lower sense of wellbeing.

As for the impact of material compensation effects on the wellbeing of rural residents, an increase in income to satisfy their physiological and psychological needs is conducive to greater wellbeing. It is undeniable that some studies have shown that increased income does not enhance happiness, which can be explained to some extent by the influence of relative income (Lu and Wang, 2020), that is the “tunnel effect”. Regarding the controversy: on the one hand, considering that the sample of this paper is rural residents, most of whom are poor in material resources and have a high desire for material compensation, satisfying their physical needs can bring happiness (Yin et al., 2021). On the other hand, this paper controls for absolute and relative incomes at the household level in the subsequent regression to minimize the impact of differences in income disparity on happiness,^5^ in order to verify whether the happiness effect of digital skills exists in the case of rural residents. This leads to the second research hypothesis of the paper.

**Hypothesis 2:** The material compensation effect of digital skills is an “inverted U”-shaped relationship, which is manifested in the fact that the mastery of digital skills can increase income and income satisfaction, and enhance the sense of wellbeing; as the level of digital skills increases, it is easy to fall into “digital traps” and “upward comparisons”, with a consequent decrease in income and income satisfaction, which in turn will reduce the sense of wellbeing.

### 3.2 Emotional immersion effects

The impact of digital skills on the spiritual dimension is often subtle, as if “immersion”, immersed in the digital world, and gradually have a positive or negative impact on their spirit, this paper focuses on the impact of digital skills on trust.

In a sociological study of Poland (Sztompka, 1999) and an economic study of public trust in rural China (Lu and Zhang, 2008), respectively, the results of these two studies show that trust is in a process of decline followed by an increase in economic (or market-oriented) transformation. Beginning with large-scale labor mobility, China's rural society has undergone a transformation from “acquaintance society” to “semi acquaintance society”, and now with the impact of the Internet, rural social trust has undergone a new round of sustained and deepening transformation, and is there a similar change in the impact of digital skills on trust in rural society?

The unfamiliarity of traditional farmers with new technologies, as well as the uncertainty of digital technologies themselves, often creates a shock of trust in the early stages of digital skill acquisition. The digital divide exacerbates this negative effect. Alesina and La Ferrara (2002) argued that people may trust people with whom they interact for longer periods of time. Therefore, as the level of digital skills increases, rural residents are able to participate in a wider range of social ways and social networks through channels such as online socialization, online entertainment and online transactions, gradually building an interpersonal network of their own and acquiring a sense of belonging to and identification with the same group as themselves, thus enhancing trust. As for the relationship between trust and wellbeing, trust is an important psychological resource and social support, and impaired trust reduces wellbeing (Delhey et al., 2011). The impact of digital skills on trust exhibits a “U”-shaped trend, initially declining and then increasing. This inference appears contradictory to the “inverted U” curve relationship posited by Hypothesis 1. Despite the establishment of new trust with increasing digital skills, a “digital trap” lurks behind the prosperity of digitalization. Its implicit destructive power on the psyche should not be underestimated. Manifestations include algorithmic capture, loss of self-value judgment, leading to digital addiction.

Addiction economics is no stranger to economic research, with economic historians conducting highly influential studies around addictive substances like tobacco and opium. From historical opium trade to the digital era of today, a new wave of addictive economics has emerged with online games, online gambling, short videos, and more. Its dependency stems from digital technology's algorithmic capture, catering to user preferences through long-term, continuous, and personalized feeds. Initially satisfying users' desires continuously, leading to stronger dependency, exacerbating digital addiction issues. Secondly, causing users to unwittingly lose self-judgment, becoming controlled by data. For example, using the number of likes to measure friendships and social connections. Ultimately, the deceptive and misleading nature of digital addiction significantly and persistently damages daily productivity and life, negatively impacting happiness. This poses an even greater threat to rural residents, many of whom already have weak information discrimination abilities.

Therefore, from the perspectives of material compensation and emotional immersion, the inverted U-shaped relationship brought by material compensation appears to have more universal significance. Trust level, as a psychologically variable emotion, can only explain certain groups. Thus, proposing the third research hypothesis in this paper.

**Hypothesis 3:** The emotional contagion effect of digital skills exhibits a “U”-shaped relationship, with trust initially decreasing and then increasing. However, the increase in trust is still insufficient to counteract the negative impact of various “digital traps” on the happiness of rural residents.

## 4 Research design

### 4.1 Data sources

This study's data is sourced from the Comprehensive Rural Revitalization Survey (CRRS) in China, covering 10 provinces/autonomous regions including Anhui, Guangdong, Guizhou, Henan, Heilongjiang, Ningxia Hui Autonomous Region, Shandong, Shaanxi, Sichuan, and Zhejiang. To ensure the representativeness and randomness of the sample, the specific sampling method includes: First, considering geographical location, socio-economic development, and rural development differences, 10 provinces/regions are selected from the eastern, central, western, and northeastern regions at a 1/3 ratio. Second, based on the per capita GDP of counties (cities, districts) in each province/region, counties (cities, districts) are divided into 5 groups, with one randomly sampled from each group using equal interval random sampling, resulting in 5 sample counties (cities, districts) per province. Third, using the same sampling method, 3 townships (towns) are sampled from each sample county (city, district), and within the township (town) samples, 2 administrative villages with varying levels of economic development are selected. Fourth, based on village lists, 12-14 rural households are randomly selected. The final survey data covers 50 counties (cities, districts), 156 townships (towns), and 300 administrative villages nationwide. The questionnaire covers aspects such as resident wellbeing and rural digitization. After excluding and handling outliers and missing values, a total of 3,433 rural household samples were obtained.

### 4.2 Variable definition

#### 4.2.1 Explained variable: rural residents' happiness

Happiness cannot be directly measured; it is commonly assessed using subjective evaluation methods. Previous studies often rely on single indicators, namely direct self-assessment of happiness, which is susceptible to momentary emotional fluctuations and may lead to inaccurate evaluations (Luo and Liu, 2022). Miret et al. (2014) categorized happiness measurement into two types: evaluative wellbeing, which assesses satisfaction with various aspects of life, and experienced wellbeing, which involves the experience of positive and negative emotions. The former reflects long-term feelings about life experiences, while the latter pertains to short-term perceptions of daily life. Veenhoven (2012) also equates life satisfaction with overall happiness, suggesting that using life satisfaction to measure happiness is the most reasonable approach. Therefore, building upon existing literature (Tan et al., 2020; Luo and Liu, 2022) and considering data availability, this study constructs a multidimensional index of life satisfaction to assess the happiness of rural residents. This approach adheres to the principles of Likert five-point scales. Specifically, it encompasses six dimensions: economic status, housing conditions, living environment, social security, village committee performance, and future life expectations. These dimensions cover economic, social, political, and psychological aspects, all closely related to the happiness of rural residents. Furthermore, Diener et al. (2013) argued that measures of life satisfaction should consider both relevance and long-term aspects. In line with this, compared to previous studies, this paper includes an additional indicator of “future life expectations” to reflect the long-term nature of happiness. The specific indicators are shown in Table 1, and a method of equal weighting of each specific indicator is adopted to compute the overall happiness index of rural residents.

**Table 1:** Comprehensive evaluation system of happiness index for rural residents.

**Variable**	**Dimensions and indicators**	**Definition and measurement**	**Mean**	**S.D**.
Rural residents' happiness	Economic conditions	Values from 1 to 5 are assigned as follows: “Very dissatisfied”, “Average”, “Not very satisfied”, “Quite satisfied”, and “Very satisfied”, indicating higher scores represent greater satisfaction.	2.57	1.05
	Housing conditions		2.33	1.01
	Living environment		2.25	1.00
	Public security		1.99	1.00
	Village committee work^a^		2.09	10.3
	Expectations for future life	Values from 1 to 5 are assigned as follows: “Very low expectation”, “Low expectation”, “Moderate”, “High expectation”, and “Very high expectation”, indicating higher scores represent greater expectations for future happiness.	4.29	0.766

^a^Refers to rural residents' evaluation of the village committee's work in their village, although they do not directly participate in the committee's activities.

#### 4.2.2 Core explanatory variable: digital skills

Regarding the definition of digital skills: (1) The UNESCO Global Framework on Digital Literacy categorizes digital competence into four dimensions: information processing, content creation, security protection, and social interaction. (2) The EU DigComp Framework 2.1 identifies five key domains of digital competence: information and data literacy, communication and collaboration, digital content creation, safety, and problem-solving. (3) China's Action Outline for Enhancing Digital Literacy and Skills of the Whole Population specifies four core dimensions: digital life, digital learning, digital work, and digital innovation. Because computer usage in rural areas is relatively limited, whereas mobile phones are cheaper, more portable, and more frequently used. Therefore, the extent of mobile phone usage better reflects the digital literacy level of rural residents. This paper uses smartphones as the medium for assessing their digital skills. Based on the above three classical frameworks, this study combines the characteristics of rural scenarios to materialize the abstract dimensions into observable cell phone usage behaviors (learning/transaction/social/entertainment).

Specifically, based on respondents' ranking of “average daily smartphone usage time” in the questionnaire, this paper distinguishes four types of skills: internet learning, internet transactions, internet socialization, and internet entertainment. Internet learning refers to using smartphones for educational purposes; internet transactions refer to using smartphones for product transactions (including mobile payment behavior); internet socialization refers to using applications like WeChat, QQ, and Weibo for social activities; internet entertainment refers to using smartphones for gaming, video watching, and reading novels, among other leisure activities. This paper constructs a comprehensive index of digital skills by summing up the number of these digital skills mastered by rural residents (Luo and Zhao, 2022). Because in this study, we focus on the improvement of the overall digital skills level of rural residents, emphasizing the measurement of the “breadth of digital exposure” rather than the “depth of digital ability”, which matches the characteristics of the rural digital access stage. Therefore, summing up the number of digital skills acquired can provide a better understanding of rural residents' ability to participate in and adapt to the digital age at a macro level. In addition, since the premise of this indicator is the “average daily smartphone usage time” in the questionnaire, the longer the usage time of a certain cell phone function usually means that the user is more skillful in that function. Therefore, to a certain extent, this indicator is able to reflect whether rural residents have actually mastered this digital skill.

#### 4.2.3 Control variables: household head characteristics, family characteristics, and village characteristics

Refer to existing studies (Knight et al., 2009; Liao, 2014; Yin et al., 2021; Luo and Liu, 2022), this paper introduces corresponding control variables at the levels of household heads, households, and villages. Household head characteristics include gender, educational level, marital status, political affiliation, and whether they hold village leadership positions. Household characteristics include family size, absolute income, and relative income. Village characteristics include village transportation conditions, economic conditions, hollowing-out phenomenon, and whether the village was formerly impoverished. Furthermore, due to differences in geographical, cultural, and resource endowments, rural residents in different regions may assess their happiness differently. This paper introduces regional dummy variables to account for these differences.

#### 4.2.4 Instrumental variable: proficiency in smartphone use

Based on the question in the questionnaire “Do you have difficulties using the functions of 4G/5G phones?”, with responses “Quite difficult, only able to make calls”, “Somewhat difficult”, and “No difficulties”, assigning values from 1 to 3 to indirectly measure the proficiency level of smartphone usage. Instrumental variables must satisfy both relevance and exogeneity requirements: Regarding relevance, since the measurement of digital skills is based on the top three functionalities of smartphone usage time, higher levels of digital skills typically imply more proficient smartphone usage, and vice versa. Additionally, this paper regresses the instrumental variable on digital skills to verify its relevance. Regarding exogeneity, on one hand, there is no direct evidence suggesting that the proficiency of smartphone usage affects personal happiness. On the other hand, this paper regresses both the digital skills variable and the instrumental variable on rural residents' happiness. If the instrumental variable affects the dependent variable only through endogenous variables, then controlling for endogenous variables should render the instrumental variable insignificant in affecting rural residents' happiness. Empirical results later in the paper demonstrate that the instrumental variable satisfies both relevance and exogeneity requirements.

#### 4.2.5 Mechanistic variables: material compensation effect and emotional immersion effect

Based on the theoretical analysis above, the material compensation effect manifests as objective income and subjective income satisfaction, measured, respectively, by responses to “Total net income of rural households last year” (logged) and “Your satisfaction with last year's household income”, rated from 1 to 5. The affective contagion effect manifests as the level of trust, measured by responses to “Do you think people around you are trustworthy?” on the questionnaire, rated from 1 to 5.

The definitions, measurements, and descriptive statistics of the above variables are shown in Table 2.

**Table 2:** Descriptive statistics of variables.

**Variable**	**Definitions and measurements**	**Mean**	**S.D**.
**Explained variable**			
Rural residents' happiness	Mean value of the six dimensions in the integrated evaluation system of indicators	2.587	0.614
**Core explanatory variable**			
Digital skills	Sum of the number of digital skills acquired	1.107	0.829
**Household head characteristics control variables**			
Gender	Male = 1; Female = 0	0.762	0.426
Educational attainment	1 = not attending school; 2 = elementary school; 3 = junior high school; 4 = high school; 5 = secondary school; 6 = vocational high school and technical school; 7 = university college; 8 = undergraduate college; 9 = postgraduate student	2.862	1.225
Marital status	Married = 1; Other = 0	0.913	0.282
Whether party member	Yes = 1; No = 0	0.256	0.436
Whether serving as a village cadre	Yes = 1; No = 0	0.195	0.397
**Household characteristics control variables**			
Household size	Number of household members	4.033	1.574
Absolute household income	Per capita household income (logarithmic)	9.401	1.116
Relative household income^a^	1 = very low; 2 = low; 3 = medium; 4 = high; 5 = very high	2.839	0.702
**Village characteristics control variables**			
Village transportation conditions	Distance of the village council from the county government (kilometers)	23.143	16.894
Village economic conditions	Disposable income per capita in villages (logarithmic)	9.444	0.546
Village hollowing out	Ratio of the number of resident population to the total household population	0.200	0.202
Ever been a poor village	Yes = 1; No = 0	0.295	0.456
**Instrumental variable**			
Proficiency in smartphone use	1 = more difficult, can only answer the phone; 2 = some difficulty; 3 = no difficulty	2.416	0.695
**Mechanism variables**			
Total net income	Total net income of head of household (logarithm)	10.713	1.152
Income satisfaction	1 = very dissatisfied; 2 = less satisfied; 3 = average; 4 = more satisfied; 5 = very satisfied	2.420	1.051
Level of trust	1 = very distrustful; 2 = not too trusting; 3 = average; 4 = more trusting; 5 = very trusting	4.251	0.747

^a^In the CRRS questionnaire, the question is “What level of income do you think your household had last year compared to the residents of your village”.

### 4.3 Preliminary analysis of samples

According to Table 2, the average happiness score of rural residents is 2.587, indicating a slightly above-average level. This suggests that rural residents in the sample are generally satisfied with various aspects of their lives and are relatively happy. The level of happiness among rural residents is comparable to studies using different data samples (Luo and Liu, 2022), reinforcing the reliability of this study's sample. The average digital skills score is 1.107, indicating that the vast majority of rural residents in the sample use at least one function such as online transactions, entertainment, or social networking. Overall, digital skills among rural residents remain at a relatively low level. According to statistics from the China Internet Network Information Center, the internet penetration rate in rural areas is 59.2%, which is moderate. This reflects that the “first-level digital divide” in rural areas has gradually narrowed, but the “second-level digital divide” remains a significant issue, highlighting the importance of digital skills.

### 4.4 Model setting

As analyzed above, there may exist a nonlinear relationship between digital skills and rural residents' happiness. Therefore, this study includes a quadratic term of digital skills to test for nonlinear effects.


(1) $$\begin{array}{l} Y_{i}=\alpha_{0}+\alpha_{1}X_{i}+\alpha_{2}X_{i}^{2}+a_{3n}Control_{i}+\varepsilon_{i} \end{array}$$

where, *Y*_*i*_ indicates the happiness of rural resident *i*; *X*_*i*_ and $X_{i}^{2}$ denote the primary and secondary terms, respectively, of the level of numerical skill of rural resident *i*; *Control*_*i*_ denotes control variables for head of household, family and village characteristics; α_0_ denotes the constant term; α_1_, α_2_, and *a*_3*n*_ represent the parameters to be estimated; ε_*i*_ is a randomized perturbation term.

## 5 Empirical results analysis

### 5.1 Benchmark regression results

This study rigorously follows the three-step method proposed by Lind and Mehlum (2010) to test for a “U”-shaped relationship. In the first step, it ensures that Equation (1) is significant and that the coefficients are in the expected direction. Table 3 reports the impact of digital skills on rural residents' happiness. The results indicate that regardless of whether control variables are included, both the linear and quadratic coefficients of digital skills are significant at the 1% level, with the linear coefficient positive and the quadratic coefficient negative, suggesting a significant inverted “U” shaped relationship between digital skills and rural residents' happiness. The second step ensures that the slopes at both ends of the data must be sufficiently steep. The third step ensures that the inflection point is within the range of the data. Through calculation, the inflection point is found to be 1.247, within the range of values for the core explanatory variable.

**Table 3:** Benchmark regression results on the impact of digital skills on the happiness of rural residents.

	**(1)**	**(2)**	**(3)**	**(4)**
Digital skills	0.099^***^	0.103^***^	0.119^***^	0.119^***^
	(0.038)	(0.038)	(0.038)	(0.038)
Digital skill^2^	−0.042^***^	−0.042^***^	−0.047^***^	−0.048^***^
	(0.016)	(0.016)	(0.016)	(0.016)
Household head characteristics		Yes	Yes	Yes
Household characteristics			Yes	Yes
Village characteristics				Yes
Regional dummy variables	Yes	Yes	Yes	Yes
Constant	2.504^***^	2.466^***^	2.822^***^	2.246^***^
	(0.024)	(0.049)	(0.100)	(0.213)
Obs	3,443	3,443	3,443	3,443
*R* ^2^	0.017	0.023	0.050	0.057

^*^, ^**^, and ^***^ denote 10%, 5%, and 1% significance levels, respectively.

Robust standard errors in parentheses, same below.

Space constraints, only regression results for the core explanatory variables are reported, and the authors can be contacted if interested in the regression results for the control variables.

Additionally, this study attempted to include a cubic term of digital skills to verify if there is an “S”-shaped relationship. The results indicate that the inclusion of higher-order terms did not improve the model fit. This further validates the reliability of the inverted “U” shaped results in this study, confirming Hypothesis 1. This illustrates that when rural residents possess certain digital skills, they can participate in digital production and life, enjoying the “digital dividends” that enhance happiness. However, as digital skills increase, it implies spending more time on smartphones and engaging with multiple applications, rural residents are highly susceptible to falling into the “digital trap”, leading to digital addiction or falling victim to online fraud, thereby reducing happiness.

### 5.2 Robustness check

As the dependent variable of rural residents' happiness in this study consists of multidimensional indicators, there may be issues of measurement error in the indicators. Furthermore, there may also be issues of omitted variable bias and endogeneity of reverse causality. To mitigate these issues, the following four methods are adopted.

#### 5.2.1 Replacement of explained variable

Considering measurement errors in indicators, the explained variable is reconstructed as follows: On one hand, this study defines rural residents' happiness as a binary variable: responses above the mean are coded as 1, and below the mean as 0 (Li, 2021). Consequently, regression becomes a linear probability model estimated using the Probit model. Despite potential information loss, the advantage of the binary approach is not needing to consider fundamental or ordinal concepts of individuals' responses to happiness. Additionally, variability in responses to certain happiness issues that are relatively low is no longer a concern. As shown in Table 4, Column (1), using the binary measurement of rural residents' happiness does not alter our conclusions. On the other hand, it is replaced with a single dimension: overall life satisfaction. Based on responses to the questionnaire item “Overall, how satisfied are you with your current life situation?” assigned values from 1 to 5. Overall life satisfaction is an ordered categorical variable, hence estimated using the ologit model. As shown in Table 4, Column (2), the relationship between digital skills and rural residents' happiness still exhibits an inverted “U” shaped curve.

**Table 4:** Estimation results of robustness check.

	**Replacement of explained variables**		**Missing variable analysis**	**Reverse causality test**
	**(1)**	**(2)**	**(3)**	**(4)**
Digital skills	0.095^***^	0.054^***^	0.113^**^	
	(0.032)	(0.002)	(0.045)	
Digital skill^2^	−0.042^***^	−0.001^***^	−0.045^**^	
	(0.013)	(0.000)	(0.019)	
Rural residents' happiness				0.021
				(0.022)
Household head characteristics	Yes	Yes	Yes	Yes
Household characteristics	Yes	Yes	Yes	Yes
Village characteristics	Yes	Yes	Yes	Yes
Regional dummy variables	Yes	Yes	Yes	Yes
Constant			2.200^***^	−1.269^***^
			(0.262)	(0.269)
Obs	3,443	3,443	2,583	3,443
*R*^2^/Pseudo *R*^2^	0.029	0.034	0.057	0.159

Columns (1) and (2) both represent marginal effects. The marginal effect in Column (2) for Y = 5 is reported, while marginal effects for other categories can be obtained by contacting the author.

^*^, ^**^, and ^***^ denote 10%, 5%, and 1% significance levels, respectively.

#### 5.2.2 Missing variable analysis

This study controlled for observable factors at the household, family, and village levels as much as possible in the baseline regression, and included regional dummy variables to control for unobservable factors at the regional level. Furthermore, since wealth can significantly influence family members' happiness, absolute and relative income at the household level were controlled for in the baseline regression. To further mitigate the impact of differences in household wealth on happiness and obtain more robust conclusions, the sample was restricted to a subsample where the baseline per capita income was below the mean of that variable. Regression results, as shown in Column (3) of Table 4, are consistent with those of the baseline regression.

#### 5.2.3 Reverse causality test

To verify whether individual digital skills among rural households are affected by their level of happiness, this study regresses rural residents' happiness on digital skill levels. As shown in Column (4) of Table 4, rural residents' happiness does not significantly influence digital skill levels, thus ruling out potential reverse causality.

#### 5.2.4 Instrumental variable approach

Despite employing various methods to mitigate endogeneity issues, this study may still be susceptible to influences from observable or unobservable factors. Therefore, this study uses proficiency in smartphone usage as an instrumental variable for digital skills. To avoid “Forbidden regression”, one-stage estimation is performed for the first and second order terms of digital skills using the first and second order terms of the instrument variables (Wooldridge, 2002; Angrist and Pischke, 2009). Columns (1) and (2) of Table 5 report the first stage regression results. It is noted that there is a correlation between the instrumental variables and the variables of interest. Column (3) of Table 5 shows that after controlling for digital skills, the instrumental variables do not significantly affect rural residents' happiness, satisfying the exogeneity requirement. Additionally, the first stage F-statistic significantly exceeds the critical value of 10. According to Stock and Yogo (2005) criteria for weak instrument tests, this confirms the effectiveness of the instrumental variable.

**Table 5:** Estimation results of the instrumental variables approach.

	**Stage I**		**Stage II**	**Exogeneity test**
	**(1)**	**(2)**	**(3)**	**(4)**
Digital skills			1.496^**^	0.091^*^
			(0.691)	(0.047)
Digital skill^2^			−0.762^**^	−0.038^**^
			(0.362)	(0.018)
IV	1.590^***^	2.735^***^		−0.019
	(0.134)	(0.304)		(0.018)
IV^2^	−0.298^***^	−0.491^***^		
	(0.030)	(0.072)		
Household head characteristics	Yes	Yes	Yes	Yes
Household characteristics	Yes	Yes	Yes	Yes
Village characteristics	Yes	Yes	Yes	Yes
Regional dummy variables	Yes	Yes	Yes	Yes
Constant			1.882^***^	2.184^***^
			(0.343)	(0.237)
Stage I *F*-value			153.33	
Obs	2,818		2,818	2,818

^*^, ^**^, and ^***^ denote 10%, 5%, and 1% significance levels, respectively.

### 5.3 Mechanism analysis

The baseline regression results above have confirmed the existence of an “inverted U” relationship between digital skills and rural residents' sense of happiness. This section further attempts to provide explanations for Hypothesis 1, namely the mechanisms behind the formation of the “inverted U” curve relationship between digital skills and rural residents' happiness. It primarily analyzes from two aspects: material compensation effect and emotional contagion effect, exploring whether income and trust are mechanisms causing the non-linear relationship between digital skills and rural residents' happiness.

#### 5.3.1 Material compensation effect

Results in columns (1) and (2) of Table 6 indicate that digital skills have an inverted U-shaped effect on income and income satisfaction, consistent with the baseline regression findings. It demonstrates that for rural residents, on the one hand, acquiring certain digital skills is beneficial for increasing their income. However, as digital skill levels rise, phenomena like digital addiction and online scams create significant harm to their lives and work, leading to decreased income. On the other hand, mastering digital skills enables rural residents to engage in digital production, gaining “digital dividends” that enhance their income satisfaction. However, as digital skill levels increase, farmers are no longer limited to a narrow reference group, leading to decreased income satisfaction due to “upward comparison”. Existing studies have largely confirmed the positive relationship between income and rural residents' happiness (Howley et al., 2017; Yin et al., 2021), indicating that changes in income can affect corresponding changes in rural residents' happiness. Thus, this validates that the inverted U-shaped relationship of the material compensation effect is an important transmission channel through which digital skills affect rural residents' happiness.

**Table 6:** Mechanism test results.

	**(1)**	**(2)**	**(3)**
	**Material compensation effects (objective)**	**Material compensation effects (subjective)**	**Emotional leaching effect**
Digital skills	0.041^***^	0.149^***^	−0.071^**^
	(0.007)	(0.004)	(0.029)
Digital skill^2^	−0.010^***^	−0.003^*^	0.022^*^
	(0.003)	(0.002)	(0.122)
Household head characteristics	Yes	Yes	Yes
Household characteristics	Yes	Yes	Yes
Village characteristics	Yes	Yes	Yes
Regional dummy variables	Yes	Yes	Yes
Constant	0.012		
	(0.043)		
Obs	3,443	3,443	3,443
*R*^2^/Pseudo *R*^2^	0.991	0.058	0.055

In columns (2) and (3), the dependent variable is an ordered categorical variable, regressed using the ologit model, and the marginal effect is the result for Y = 5. The remaining categorical results for the marginal effect can be obtained by contacting the authors. ^*^, ^**^, and ^***^ denote 10%, 5%, and 1% significance levels, respectively.

#### 5.3.2 Emotional contagion effect

Results in column (3) of Table 6 indicate that digital skills have a U-shaped curve relationship with trust, meaning trust initially decreases and then increases, consistent with existing research findings (Sztompka, 1999; Lu and Zhang, 2008). However, this is inconsistent with the inverted U-shaped curve relationship between digital skills and rural residents' happiness. The explanation for this is that although trust shows a positive impact as digital skill levels increase, on the one hand, this increase in trust is not sufficient to counterbalance the negative impacts of decreased income and income satisfaction. On the other hand, trust-building is slow but can fracture rapidly, thus the negative impact of decreased trust on happiness is highly subtle and enduring. Existing research has also validated the relationship between trust trauma and decreased happiness (Tokuda and Inoguchi, 2008; Delhey et al., 2011). Identifying and measuring these negative effects will be the focus of future research in this paper.

### 5.4 Heterogeneity analysis

#### 5.4.1 Differences in income levels

The preceding analysis has confirmed that income serves as a crucial mechanism leading to an inverted U-shaped relationship between digital skills and rural residents' happiness. Does this relationship persist across different income levels? This study divides the sample equally into low, medium, and high income groups based on total income of farmers. As shown in Table 7.

**Table 7:** Results of heterogeneity analysis of differences in income levels.

	**(1)**	**(2)**	**(3)**
	**Low income level**	**Middle income level**	**High income level**
Digital skills	0.131^*^	−0.006	0.191^***^
	(0.073)	(0.070)	(0.066)
Digital skill^2^	−0.044	−0.006	−0.072^***^
	(0.035)	(0.029)	(0.024)
Household head characteristics	Yes	Yes	Yes
Household characteristics	Yes	Yes	Yes
Village characteristics	Yes	Yes	Yes
Regional dummy variables	Yes	Yes	Yes
Constant	1.797^***^	2.438^***^	2.298^***^
	(0.382)	(0.772)	(0.469)
Obs	1,154	1,142	1,147
*R*^2^/Pseudo *R*^2^	0.061	0.080	0.059

^*^, ^**^, and ^***^ denote 10%, 5%, and 1% significance levels, respectively.

In the low-income group, there is no inverted U-shaped relationship between digital skills and rural residents' happiness; only the linear term is significantly positive. For farmers with low income, digital skills bring them material or spiritual rewards, enjoying the benefits of the “digital dividend”, which provides satisfaction and reflects their contentment and “sufficiently rich and at ease” attitude toward life.

In the medium-income group, digital skills are not significant.

In the high-income group, results consistent with the baseline regression are obtained, and the absolute values of the coefficients for the linear and quadratic terms are greater than those in the baseline regression, indicating that for high-income farmers, both the positive impact of the “digital dividend” and the negative impact of the “digital trap” are enhanced. The explanation for this is two-fold: on one hand, the digital economy exhibits a multiplier effect; farmers with high incomes have a strong economic foundation, and improvement in digital skills can lead to a stronger positive material compensation effect. On the other hand, a stronger economic foundation is more susceptible to addictive economies and deceived by false information, often resulting in greater economic losses.

#### 5.4.2 Differences in education levels

Due to varying educational levels among different individuals, there may be disparities in their ability to reap the benefits of digital skills. Therefore, this paper further examines the educational heterogeneity at the individual level to explore the differences in digital welfare resulting from individual disparities. In light of the current educational situation in rural China, individuals with no schooling or only primary-school education are classified as having a low educational level, those with junior-high-school education are considered to have a medium educational level, and those with senior-high-school education or above are regarded as having a high educational level.

As shown in Table 8, indicate that the mastery of digital skills does not significantly enhance the wellbeing of the highly educated group. Instead, it has a more significant impact on the wellbeing of the medium and low-educated groups. Moreover, the probability of wellbeing improvement is higher for the low-educated group than for the medium-educated group. This suggests that the mastery of digital skills can effectively narrow the gap between different groups caused by education, generating a welfare effect.

**Table 8:** Results of heterogeneity analysis of differences in educational levels.

	**(1)**	**(2)**	**(3)**

	**Low educational level**	**Middle educational level**	**High educational level**
Digital skills	0.121^*^	0.103^*^	−0.005
	(0.069)	(0.058)	(0.097)
Digital skill^2^	−0.052^*^	−0.036	−0.021
	(0.031)	(0.024)	(0.034)
Household head characteristics	Yes	Yes	Yes
Household characteristics	Yes	Yes	Yes
Village characteristics	Yes	Yes	Yes
Regional dummy variables	Yes	Yes	Yes
Constant	2.282^***^	2.162^***^	2.757^***^
	(0.395)	(0.306)	(0.528)
Obs	1,287	1,549	607
*R*^2^/Pseudo *R*^2^	0.045	0.079	0.078

^*^, ^**^, and ^***^ denote 10%, 5%, and 1% significance levels, respectively.

## 6 Discussion

Our discovery of the “inverted U” curve between digital skills and rural happiness enriches digital—wellbeing knowledge. It challenges the idea that more digital skills always mean more happiness. Similar to past tech—adoption studies, we find a threshold. Initial digital use can boost economic and social aspects, but over-reliance may cause harm. The mechanism analysis, highlighting the material compensation effect as the main driver of the “inverted U” shape, offers a new framework. It combines digital skills with economic factors like income, showing their complex link. Although emotional immersion has a positive side, it can't counterbalance the negative material effect, shedding light on the balance between objective and subjective factors in happiness.

For digital village construction and rural policies, the research matters. The digital dividend benefits both low-and high-income farmers, stressing the need for digital skills training. However, high—income farmers are at risk of the “digital trap”. Thus, digital literacy and healthy-use education, such as avoiding digital addiction, should be provided. Low-income farmers, with a positive linear relationship between skills and happiness, deserve more resources for skill improvement, like targeted training and better digital access. Also, as digital skills can reduce the education-based welfare gap, integrating digital education into rural strategies can enhance overall wellbeing.

## 7 Conclusions and suggestions

This paper analyzes the impact of digital skills on rural residents' happiness based on 2020 CRRS data, with the following conclusions:

(1) Baseline results indicate an inverted U-shaped relationship between digital skills and rural residents' happiness. This manifests as enjoying the “digital dividend” and enhancing happiness with a certain level of digital skills; however, as digital skill levels increase, individuals are prone to falling into the “digital trap”, which reduces happiness.
(2) Mechanism analysis reveals that the effect of digital skills on rural residents' happiness, initially promoting and then inhibiting, operates through a material compensation effect, manifested as an initial increase followed by a decrease in income and income satisfaction. Despite the positive impact shown by emotional contagion effects, they are insufficient to counterbalance the negative impact of material compensation effects.
(3) Heterogeneity analysis indicates that the impact of digital skills on rural residents' happiness is significant in both low-income and high-income samples. However, high-income farmers are similarly susceptible to stronger negative effects, showing a non-linear inverted U-shaped relationship. Low-income farmers experience only positive effects, demonstrating a linear relationship. Besides, Mastery of digital skills has a more significant effect on the wellbeing of the lower and middle educated groups, and is effective in bridging the gap between the different groups.

The research findings of this article have certain policy implications for enhancing the happiness of rural residents and building beautiful and prosperous countryside, as well as promoting rural revitalization. Additionally, they contribute to the implementation of the Digital China initiative in rural areas.

Firstly, enhancing rural residents' digital skills and strengthening the “digital dividend” happiness effect, especially for low-income groups in rural areas. It is necessary to both overcome bottlenecks and break through difficulties: on one hand, aligning with the Digital China initiative, focus should be placed on advancing digital rural construction, providing essential digital infrastructure guarantees for vulnerable rural groups, and eliminating hard obstacles to rural residents' access to digital technology. On the other hand, based on rural production and life realities, promote literacy training in information technology, cultivate farmers' initiative in learning digital skills, and overcome cognitive and volitional barriers among rural residents to participate in digital production and life. This will harness the positive impact of digital skills on material compensation, increase rural residents' actual income, and enhance their happiness.

Secondly, creating a clear rural cyberspace to mitigate the negative impact of the “digital trap”, especially for high-income groups in rural areas. This requires dual efforts from external drivers and internal awareness: on one hand, through compulsory measures, rural network inspection and supervision should be strengthened. By conducting household visits, disseminating mobile announcements, and enhancing farmers' awareness and skills in network security, we can promptly curb the spread of various false or negative online information, thereby avoiding the negative impact on material compensation effects and trust erosion that may lead to reduced happiness. On the other hand, through self-regulation, emphasis should be placed on guiding internet culture, focusing on high-quality content and correct values in online information, fostering positive spiritual influences of digital skills, shaping good physical and psychological health, and ultimately enhancing happiness.

## Data Availability

The original contributions presented in the study are included in the article/supplementary material, further inquiries can be directed to the corresponding author.

## Footnotes

^1^Happiness is often considered to be subjective wellbeing, and Veenhoven (2012) refers to life satisfaction as a synonym for general happiness. Therefore, in this paper, “happiness”, “subjective wellbeing”, and “life satisfaction” are regarded as equivalent concepts and are used interchangeably in the text. In addition, the terms “rural residents”, “farmers”, and “farm households” are used interchangeably.

^2^The “secondary digital divide” refers to the differences in the skills of applying digital technology between different regions and groups, also known as the skills divide (Chen and Zhou, 2022), which corresponds to the digital skills in this paper.

^3^It refers to a digital technology effect that recognizes the content you may be interested in within a short period of time and continuously pushes relevant content to capture users through interaction design and user-friendly recommendation algorithms.

^4^ https://www.sohu.com/a/455663853_161795

^5^All samples in this paper are restricted to those with a source of income.
